# *AGK‐BRAF* gene fusion is a recurrent event in sporadic pediatric thyroid carcinoma

**DOI:** 10.1002/cam4.698

**Published:** 2016-03-31

**Authors:** Maria Isabel C. V. Cordioli, Lais Moraes, Gianna Carvalheira, Luiza Sisdelli, Maria Teresa S. Alves, Rosana Delcelo, Osmar Monte, Carlos A. Longui, Adriano N. Cury, Janete M. Cerutti

**Affiliations:** ^1^ Genetic Bases of Thyroid Tumors LaboratoryDivision of GeneticsDepartment of Morphology and GeneticsUniversidade Federal de São PauloSão PauloSPBrazil; ^2^ Department of PathologyUniversidade Federal de São PauloSão PauloSPBrazil; ^3^ Department of PediatricsIrmandade da Santa Casa de Misericórdia de São PauloSão PauloSPBrazil; ^4^ Department of MedicineIrmandade da Santa Casa de Misericórdia de São PauloSão PauloSPBrazil

**Keywords:** *AGK‐BRAF*, BRAF V600E, papillary thyroid carcinoma, pediatric thyroid cancer, sporadic thyroid carcinoma

## Abstract

Thyroid cancer is the fastest increasing cancer worldwide in all age groups. Papillary thyroid carcinoma (PTC) is the most common type of thyroid cancer in both adults and children. PTC genomic landscape has been extensively studied in adults, but information regarding sporadic pediatric patients is lacking. Although BRAF V600E mutation is highly prevalent in adults, this mutation is uncommon in pediatric cases. As adult and pediatric PTC is a mitogen‐activated protein kinase‐driven cancer, this altered pathway might be activated by different genetic events. The aim of this study was to investigate the occurrence of *AGK‐BRAF* fusion gene, recently described in radiation‐exposed pediatric PTC, in a cohort of exclusively sporadic pediatric PTC. The series consisted of 30 pediatric PTC younger than 18 years of age at the time of diagnosis and 15 matched lymph node metastases (LNM). Primary tumors and matched LNM were screened for the presence of the *AGK‐BRAF* fusion transcript by RT‐PCR. To confirm the identity of the amplified products, randomly selected samples positive for the presence of the fusion transcripts were sequenced. Moreover, *BRAF* dual‐color, break‐apart probes confirmed *BRAF* rearrangement. Overall, the *AGK‐BRAF* fusion gene was detected in 10% (3/30) of primary tumors. For one of these cases, paired LNM was also available, which also shows the presence of *AGK‐BRAF* fusion gene. This study described, for the first time, the presence of *AGK‐BRAF* in sporadic pediatric PTC. Understanding the molecular events underlying pediatric PTC may improve preoperative diagnosis, allow molecular prognostication and define a therapeutic approach toward sporadic PTC patients.

## Introduction

An increasing incidence of thyroid cancer has been reported in most populations worldwide 1, 2. Thyroid cancer is the fifth most common cancer in women in the United States, accounting for approximately 5% of all cancer 3. Recently, a rise in thyroid cancer incidence rate has also been reported in pediatric patients, mainly among adolescents 3, 4. In fact, thyroid cancer is the second most prevalent cancer in females with 15–19 years of age 5. Similar to adults, the great majority of pediatric follicular cell‐derived thyroid carcinomas are papillary thyroid carcinomas (PTC), with nearly 75–90% of cases 6.

The clinical presentation and outcomes of thyroid carcinoma differ between pediatric and adult population. Although pediatric patients are more likely to present a more advanced stage of disease at diagnosis and higher risk of recurrent and persistent disease than adults, they usually have an excellent overall survival 7, 8. Furthermore, a great heterogeneity within the pediatric group has been reported. Some studies have suggested worse outcome for children compared to adolescents 9, 10.

It is still unclear whether the clinicopathological differences observed between pediatric and adult population may be due to the existence of distinct genetic alterations. In fact, the frequency and spectrum of mutations in adult PTC is markedly different than that in pediatric PTC 11, 12. Some studies have also reported a different spectrum of mutations within pediatric group. Actually, the prevalence of the BRAF V600E mutation, the most common genetic event found in adult PTC 13, is significantly lower in sporadic and radiation‐exposed pediatric PTC 14, 15, 18. On the other hand, a high prevalence of genetic rearrangements has been described in both sporadic 11, 16 and radiation‐exposed pediatric thyroid carcinomas 17, 18.

Interestingly*, BRAF* rearrangements, in which the BRAF kinase domain is fused to a variety of 5′ partners, have been reported in several solid tumors types 19 as well as in PTC 20. The new fusion gene (*AKAP9‐BRAF*) was found in radiation‐exposed PTCs and results from an in‐frame fusion of the exons 1–8 of the *AKAP9* gene (A‐kinase anchor protein 9) to exons 9–18 of *BRAF* gene 20.

As *BRAF* fusion represent an alternative mechanism of BRAF activation, one could hypothesize that *BRAF* could be activated in pediatric PTCs through rearrangement. In fact, recently, *AGK‐BRAF* rearrangement was described in one case of radiation‐exposed pediatric PTC, but was not identified in pediatric cases from patients with unknown radiation exposure 18. This rearrangement was caused by an inversion of the long arm of chromosome 7, which juxtaposes the exons 1 and 2 of the *AGK* (acylglycerol kinase) to exons 8–18 of *BRAF* 18. The expression of this fusion oncogene promotes a constitutive activation of MEK and ERK phosphorylation, thus activating the mitogen‐activated protein kinase (MAPK) cascade 18. *AGK‐BRAF* was later described in one PTC from adult patient with apparently no history of radiation exposure 21.

To elucidate alternative mechanisms of aberrant *BRAF* activation*,* this study investigated the presence of *AGK‐BRAF* fusion oncogene in a cohort of predominantly sporadic pediatric PTC.

## Material and Methods

### Thyroid Samples

The series consists of 45 formalin‐fixed paraffin‐embedded (FFPE) sections from 30 primary PTC and 15 matched lymph node metastases (LNM) from patients who underwent thyroid surgery from 1993 through 2012 at Hospital São Paulo (Universidade Federal de São Paulo) and Hospital da Santa Casa de São Paulo. All samples were reviewed by two pathologists (RD and MTSA). As recommended by the ATA guidelines, all pediatric patients included in this study were ≤18 years of age at the time of diagnosis 22. The study was conducted under the approval of the Review Boards and Research Ethical Committees of the affiliated institutions.

### RNA isolation and cDNA synthesis

Total RNA was isolated from 10‐*μ*m thick FFPE sections using the Recover All Total Nucleic Acid isolation kit (Ambion Inc., Austin, TX). Total RNA (500 ng) was treated with DNAse and reverse‐transcribed into cDNA with both 50 *μ*M oligo(dT)_12‐18_ and 50 ng random hexamers using a Superscript III transcriptase kit (Invitrogen Corp., Carlsbad, CA).

### Transient transfection of *AGK‐BRAF* fusion gene in thyroid cells

FTC 238 thyroid carcinoma cells, established from a lung metastases of a human follicular thyroid carcinoma, purchased from the European Collection of Cell Cultures (ECACC, Health Protection Agency, Salisbury, UK) were cultured in Dulbecco's modified essential medium (DMEM):Ham's F12 (1:1) medium supplemented with 5% fetal bovine serum (FBS) (Life Technologies, Carlsbad, CA). FTC 238 cells were transiently transfected with 10 *μ*g of pLVX‐*AGK‐BRAF* plasmid by electroporation using a Gene Pulser II (Bio‐Rad Laboratories Inc., Hercules, CA). The oncogene‐transfected cells were harvested, and the total RNA was isolated using TRIzol Reagents (Invitrogen Corp.) and reverse‐transcribed into cDNA, as above mentioned. The cDNA generated from cells expressing the fusion transcripts was used as a positive control. The pLVX‐*AGK‐BRAF* plasmid was kindly donated by Dr. James Fagin (Memorial Sloan‐Kettering Cancer Center).

### Detection of *AGK‐BRAF* fusion transcript

All of the samples were screened for the presence of *AGK‐BRAF* fusion transcript by RT‐PCR, as previously described 18. Briefly, cDNA (2 *μ*L) was subjected to PCR amplification using 1.0 unit Platinum Taq DNA Polymerase (Invitrogen Corp.) and 2 pmol of each primer, as described. The samples were incubated at 95°C for 10 min and then subjected to 40 cycles of denaturation at 95°C for 30 sec, annealing at 60°C for 30 sec, and polymerization at 72°C for 30 sec, with a 5‐min final extension at 72°C. The efficiency of cDNA synthesis was tested using *RPS8* as internal control, as previously described 23. A positive and negative control was included in each real‐time PCR run. The PCR products were resolved on a 2% agarose gel and visualized on a Bio‐Rad Gel Doc EZ system (Bio‐Rad). To confirm the identity of the amplified products, positive samples were sequenced using the BigDye Terminator Cycle Sequencing Kit (PE Applied Biosystems, Foster City, CA). The primers used to detect the *AGK‐BRAF* fusion transcript, located in exon 2 of *AGK* and exon 8 of *BRAF*, were previously described and validated 18.

### Dual‐color break‐apart fluorescence *in situ* hybridization (FISH)

A commercially available dual‐color, break‐apart assay was used to test possible breakage of *BRAF* gene resulting from structural rearrangements. The two differentially labeled probes, flanking the *BRAF* gene, were cohybridized in two *AGK‐BRAF*‐positive PTCs. Targeted tumor areas were circled, following review of the corresponding H&E slide by a pathologist (RD), prior to the FISH assay. A 3‐*μ*m thick unstained tissue sections were deparaffinized, rehydrated, and incubated in the pretreatment solution and washed according to manufacturer's protocol (DAKO, Glostrup, Denmark). Slides were then incubated with 5 *μ*L solution containing the labeled FISH *BRAF* probes and IQFISH Fast Hybridization buffer (SureFISH break‐apart probes; Agilent Technologies, Palo Alto, CA) denatured at 80°C for 10 min and hybridized overnight at 37°C. Posthybridization wash was performed in stringent wash buffer at 65°C and two nonstringent washes at room temperature. The slides were then mounted with 10 *μ*L of Mounting Buffer with 4′,6‐diamidino‐2‐phenylindole (DAPI) as a counterstaining. The FISH results were evaluated with fluorescent microscope Zeiss (Zeiss, Oberkochen, Germany) using ISIS Karyotype Image System (Metasystems, Altlussheim, Germany). At least 100 nonoverlapping and intact nuclei were evaluated.

## Results

### Clinical and pathological features

We systematically investigated the prevalence of *AGK‐BRAF* mutation in all primary tumors and matched LNM. Age ranged from 4 to 18 years old (mean = 11.36 years). Twenty‐one patients (70%) were females and 9 (30%) were males. The study included 12 classical PTC (CPTC), 12 follicular variant of PTC (FVPTC), four diffuse sclerosing variant of PTC (DSVPTC), and two other variants of PTC. The clinical and pathological features evaluated are summarized in Table 1.

**Table 1:** Summary of the clinicopathological features and occurrence of AGK‐BRAF fusion oncogene in pediatric thyroid carcinoma

Case	PTC Variant	Age (years)	Gender	Tumor Size (cm)	Multifocality	Lymph node Metastasis	Distant Metastasis	Extrathyroidal extension	TNM	Radiation Exposure	*AGK‐BRAF*
1	Classical	7	M	1.4	No	Yes	No	No	T1N1M0	No	No
2^a^	Follicular	18	F	4.5	No	Yes	No	NA	T3N1M0	No	No
3	Classical	4	M	1.7	No	Yes	Yes	Yes	T4N1M1	No	No
4	Classical	13	F	3.2	No	No	No	No	T2N0M0	No	No
5^a^	Diffuse Sclerosing	17	F	1.5	Yes	Yes	No	No	T1N1M0	No	No
6	Classical	4	M	0.7	No	Yes	No	Yes	T3N1M0	No	No
7^a^	Classical	18	F	3.5	Yes	Yes	No	No	T2N1M0	No	No
8	Follicular	17	F	2.5	No	No	No	No	T2N0M0	No	No
9^a^	Classical	7	F	6	Yes	Yes	Yes	Yes	T4N1M1	No	No
10^a^	Follicular	12	M	3	Yes	Yes	Yes	Yes	T4N1M1	No	No
11	Classical	5	F	3	No	Yes	Yes	Yes	T4N1M1	No	No
12	Diffuse Sclerosing	13	F	2.5	Yes	Yes	No	No	T2N1M0	No	No
13^a^	Diffuse Sclerosing	9	M	1.7	Yes	Yes	Yes	Yes	T4N1M1	No	No
14^a^	Classical	18	F	3.5	No	Yes	No	No	T2N1M0	No	No
15	Follicular	12	F	1.8	No	No	No	No	T1N0M0	No	No
16	Classical	12	F	NA	Yes	Yes	Yes	Yes	T4N1M1	No	No
17	Follicular	6	F	NA	NA	Yes	No	NA	TxN1M0	No	No
18^a^	Follicular	13	M	2	Yes	Yes	No	Yes	T4N1M0	No	No
19	Encapsulated	10	F	2	No	No	No	No	T1N0M0	No	No
20^a^	Classical	15	M	4.5	Yes	Yes	Yes	Yes	T4N1M1	No	Yes
21^a^,^b^	Follicular	13	F	5	Yes	Yes	Yes	Yes	T4N1M1	No	Yes
22^a^	Classical	16	F	2	Yes	Yes	No	No	T1N1M0	No	No
23^a^	Follicular	8	F	2.5	No	Yes	No	No	T2N1M0	No	No
24^a^	Solid	8	M	0.9	Yes	Yes	Yes	Yes	T4N1M1	No	No
25^a^	Diffuse Sclerosing	9	F	2.1	No	Yes	No	Yes	T4N1M0	No	No
26	Classical	7	F	1.6	No	No	No	Yes	T3N0M0	No	Yes
27^a^	Follicular	6	F	3.5	Yes	Yes	Yes	Yes	T4N1M1	No	No
28	Follicular	14	F	1.7	No	No	No	No	T1N0M0	No	No
29	Follicular	18	F	1	No	Yes	No	No	T1N1M0	No	No
30	Follicular	12	M	2.2	Yes	Yes	No	No	T2N1M0	No	No

a PTC samples with matched and Lymph node metastasis.

b PTC samples with matched Lymph node metastasis which was positive for AGK‐BRAF.

### Recurrent *AGK‐BRAF* rearrangement in sporadic pediatric PTCs

**Figure 1:**
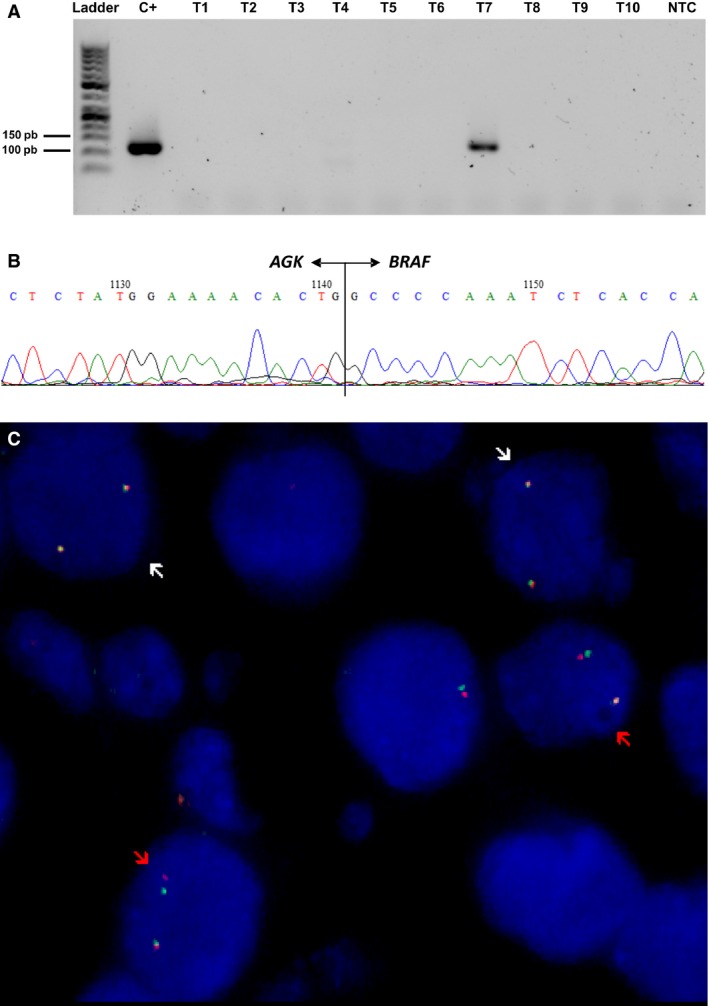
Screen for the presence of *AGK‐BRAF* fusion oncogene in sporadic pediatric PTC. Representative results of RT‐PCR analysis performed in sporadic pediatric PTC (T1‐T10). Positive (C+) and negative controls (NTC) were included in each run. Positive cases showed the proper size range (113 bp), as showed in C+ and case 20 (T7) (A). Sanger sequencing confirmed the presence of *AGK‐BRAF* fusion oncogene (B). Dual‐color, break‐apart FISH confirmed the breakage of *BRAF* gene resulting from structural rearrangements *BRAF* (C). Nuclei exhibiting rearrangement showed the presence of one red and green split signals (red arrow), in addition to the fused yellow or red‐green signal (white arrow).

In order to optimize RT‐PCR reaction, we primarily used cDNA obtained from FTC cells transiently transfected with plasmid containing the *AGK‐BRAF* fusion gene. Different primer concentrations and PCR conditions were assayed. AGK‐BRAF was found in two primary tumors. Moreover, in one patient *AGK‐BRAF* rearrangement was identified in the LNM, while the fusion gene was not observed in the paired primary tumor. As this patient presented a multifocal PTC (case 21), additional foci were selected for further investigation. One out of five foci presented the *AGK‐BRAF* fusion oncogene. Overall, the *AGK‐BRAF* fusion gene was found in nearly 10% (3/30) of primary tumors and in about 6% (1/15) of LNMs (Table 1; Fig. 1). The presence of *AGK‐BRAF* fusion oncogene was confirmed by sequencing analysis (Fig. 1).

Additionally, two cases that were positive for *AGK‐BRAF* by RT‐PCR were selected to test possible breakage of the *BRAF* gene and, therefore, to confirm the *BRAF* rearrangement. In addition to the fused yellow or red‐green signal, the selected fields showed the presence of red and green split signals (Fig. 1). The split‐apart *BRAF* signal was identified in 30% and 36% of cells. Nearly 70% of cells exhibited only two fused yellow or red‐green signals, confirming tumor heterogeneity.

### *AGK‐BRAF* fusion oncogene and clinical‐pathological features of sporadic pediatric PTC

Among three patients positive for *AGK‐BRAF*, the mean age at diagnosis was 11.66 years (range 7–15 years). Two tumors with *AGK‐BRAF* fusion were of classical histology and one of follicular variant. Extrathyroidal extension was observed in all patients with *AGK‐BRAF* rearrangement. The prevalence of patients with distant metastasis at diagnosis and multifocality was higher in the AGK‐BRAF‐positive groups than in AGK‐BRAF‐negative groups (Table 2).

**Table 2:** Pediatric PTC characteristics according to the prevalence of *AGK‐BRAF*

	Total No.(*n *= 30)	*AGK‐BRAF* Negative No (%)(*n *= 27)	*AGK‐BRAF* Positive No (%)(*n *= 3)
Age ± SD (mean/years)	11.36	11.33	11.66
Tumor size ± SD (mean/cm)	2.55	2.41	3.7
Gender			
Female	21	19 (70)	2 (66)
Male	9	8 (30)	1 (34)
Extrathyroidal extension	14	11 (40)	3 (100)
Multifocal disease	14	12 (44)	2 (66)
LN metastases	24	22 (81)	2 (66)
Distant metastases	10	8 (29)	2 (66)

## Discussion

The Cancer Genome Atlas (TCGA) Research Network, using different platforms combined with clinicopathological data, characterized the genomic landscape of nearly 500 PTCs of adults. The study confirmed that PTC is a MAPK‐driven cancer, identified new cancer‐causing gene mutations, as well as new fusion transcripts and somatic copy number alteration. These findings reduced the so‐called “dark matter” of the PTC. Importantly, the large collection of genetic alterations, combined with a comprehensive transcriptomic and proteomic analysis, exposed fundamental biological variances between PTCs. This increased knowledge helped to stratify PTC into subgroups, which eventually will improve preoperative diagnosis of thyroid nodules, prognosis, and treatment of adult PTC 21.

Despite intensive efforts, much less is known about the genetic alterations that are, in fact, “driver genes” in pediatric PTC. Although pediatric PTCs are also a MAPK‐driven cancer, the spectrum of mutations differs between adults and pediatric tumors. Furthermore, radiation‐exposed and sporadic pediatric PTCs likely have different genetic landscapes. In fact, Nikiforov et al. 17, provided the first evidence that they have different molecular signature. The authors reported that the prevalence of *RET/PTC* rearrangements is markedly different between sporadic and radiation‐exposed pediatric PTC. Not only the overall prevalence of *RET/PTC* diverges between sporadic and radiation‐exposed PTC but also the prevalence of *RET/PTC3* isoform was higher than *RET/PTC1* in radiation‐exposed cases 12, 17.

Recently, another group described that the proportion of samples harboring fusion oncogenes in radiation‐exposed pediatric PTC is markedly higher (85%) from that seen in nonradiation‐exposed group (33%), while point mutations have been mainly found in nonradiation‐exposed patients than in radiation‐exposed PTC patients 18

Most of the efforts to determine the landscape of pediatric cases have been concentrated in radiation‐exposed pediatric thyroid cancer, while in the routine most cases of thyroid cancer are actually sporadic cases.

The molecular differences between adult and pediatric PTC and the fact that fewer genetic events were described in pediatric PTC may impact on the utility of molecular testing for diagnosis of thyroid nodules in children. In fact, the ATA Guidelines for Children with thyroid nodules and differentiated thyroid cancer suggested that, although in adults molecular testing aids in the management of thyroid nodules with indeterminate cytopathology, insufficient data exist in children to rely on negative genetic studies. Therefore, the test cannot be recommended in routine clinical on pediatric patients practice until further studies are conducted 22.

This study identified the presence of *AGK‐BRAF* fusion gene in sporadic pediatric PTC. Although BRAF V600E mutation is uncommon in both radiation‐exposed and sporadic pediatric PTC 12, 15, 24, our findings reveal that BRAF fusion might be an alternative mechanism of MAPK pathway activation. It has been previously demonstrated that expression of *AGK‐BRAF* in NIH3T3 and COS‐7 cells promotes constitutive activation of MAPK signaling pathway and induces NIH3T3 cell growth and colony formation 18.

The frequency of *BRAF* fusion in this cohort of sporadic cases, one of the largest of literature, was validated using different approaches. FISH analysis allowed us to detect clonal rearrangements and to ratify tumor heterogeneity. Finally, dual‐color, break‐apart *BRAF* probe will help us to detect the presence of any fusion within the *BRAF* gene in sporadic pediatric cancer.

It has been suggested that biological differences may explain the clinical and pathological features differences between pediatric and adult patients. It still remains unclear whether *AGK‐BRAF* correlates with clinicopathological parameter in PTC such as age, presence of metastases, histological subtypes, and advanced clinical stages. In our study, *AGK‐BRAF* fusion appears to be related to a more aggressive biological behavior, as extrathyroidal extension was seen in all patients with *AGK‐BRAF* rearrangement. Additionally, multifocality, lymph node, and distant metastasis at diagnosis were observed in two patients out of three patients with *AGK‐BRAF* rearrangement. Unfortunately, no clinical information is available for the previously described *AGK‐BRAF*‐positive radiation‐exposed PTC 18. Nevertheless, further analysis, ideally multicenter studies, is needed to confirm this hypothesis and to better elucidate the biological behavior of sporadic pediatric PTC with *AGK‐BRAF* fusion gene.

In summary, our findings provide additional insight to our current understanding of tumor biology of sporadic pediatric PTC. Further efforts should be undertaken to define the genomic landscape of sporadic pediatric PTC. The knowledge of the molecular events underlying this group of patients would be extremely useful to improve the accurate diagnosis of thyroid nodules and prevent unnecessary thyroid surgeries, allow molecular prognostication and define a therapeutic approach toward sporadic PTC patients.

## Conflict of Interest Statement

The authors have reported no conflicts of interest.
